# Systematic Parameterization of Ion–Surfactant Interactions in Dissipative Particle Dynamics Using Setschenow Coefficients

**DOI:** 10.1021/acs.jpcb.2c00101

**Published:** 2022-03-15

**Authors:** Ennio Lavagnini, Joanne L. Cook, Patrick B. Warren, Christopher A. Hunter

**Affiliations:** † Department of Chemistry, University of Cambridge, Lensfield Road, Cambridge CB2 1EW, U.K.; ‡ Unilever R&D Port Sunlight, Quarry Road East, Bebington CH63 3JW, U.K.; § STFC Hartree Centre, Sci-Tech Daresbury, Warrington WA4 4AD, U.K.

## Abstract

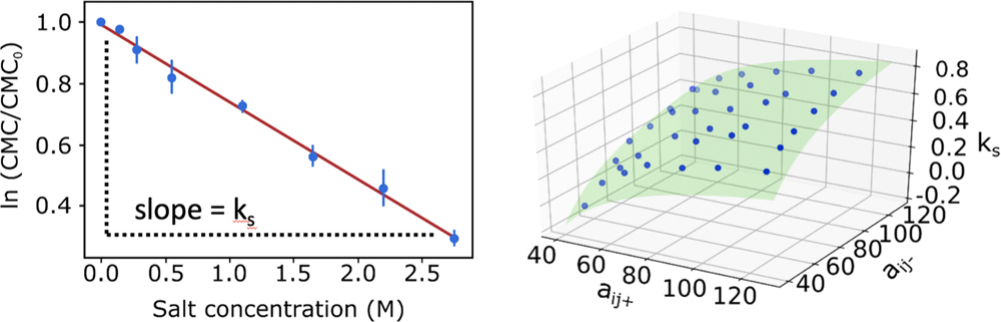

Dissipative particle dynamics (DPD) simulations of nonionic surfactants with an added salt show that the Setschenow relationship is reproduced; that is, the critical micelle concentration is log-linearly dependent on the added salt concentration. The simulated Setschenow coefficients depend on the DPD bead–bead repulsion amplitudes, and matching to the experimentally determined values provides a systematic method to parameterize the interactions between salt ion beads and surfactant beads. The optimized ion-specific interaction parameters appear to be transferrable and follow the same trends as the empirical Hofmeister series.

## Introduction

The presence of ions in a solution affects numerous chemical and biophysical phenomena. The magnitude of these effects often follow ion-specific trends such as the Hofmeister series,^1^ which was initially introduced to rank the propensity of salts to decrease the solubility of proteins (salting out) and was subsequently discovered to hold for other phenomena such as partitioning between two liquid phases,^2,3^ macromolecular conformational transitions,^4^ enzyme activity,^5^ protein denaturation,^6,7^ viscosity, and critical micelle concentrations (CMCs) of surfactant solutions.^8−10^ For a nonelectrolyte, the salting out effect is described by the Setschenow equation^11^
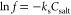
1which describes how the activity coefficient *f* of an uncharged solute depends on the salt concentration *C*_salt_. In this, *k*_s_ is an empirical, salt-specific Setschenow coefficient. We note that the Setschenow relation can be expressed in terms of natural or base 10 logarithms; for this work, we use the natural logarithm “ln”. Also, regarding the level of accuracy to which we are working, it is not necessary to distinguish between molar and molal salt concentrations in eq 1 since the difference typically amounts to only a few percent for concentrations less than 1 M at room temperature and pressure. Moreover, since the Setschenow coefficients are arguably defined by their limiting values in eq 1 as *C*_salt_ tends to zero, this obviates the need to consider the densities of the salt solutions.

While the accurate determination of Setschenow coefficients relies on experimental methods, several models have been developed to predict the effect of a particular salt on a molecule in solution. Among these, an early attempt was made by Debye and MacAulay;^12^ then, McDevit and Long later correlated *k*_s_ for benzene, as a solute, to the change in the volume of the solvent when salt is added;^13^ Conway *et al.* used dielectric saturation to extend the theory to polyions;^14^ and Masterton and Lee adopted a scaled particle theory to derive a general expression for the salt effect on benzene derivatives.^15^ The use of empirical parameters and the relatively low accuracy of these models limit their use to only a few systems.^16^ Wen-Hui *et al.* linked the Setschenow coefficient for sodium chloride to the Le Bas volume (*V*_LB_) through a simple linear correlation, *k*_s_ = 0.0018 *V*_LB_,^17^ while Gould used the intrinsic solubility of the solute.^18^ Ni *et al.* showed how a linear correlation with the partition coefficient of the solute outperformed previous models,^19,20^ but data availability limited the study to sodium chloride. More recently, Zhou^21^ developed an electrostatic theory to describe the interaction of macromolecules with salt ions, showing good correlation with experimental data for protein solubility and stability. The salting out effect can be directly linked to an increase in surface tension, and Li *et al.* studied the change in solvation free energy of small molecules in the presence of different salts, concluding that Setschenow coefficients can be explained by the formation of nonpolar cavities in the salt solution and are not due to the direct interaction between solutes and ions.^22^

For surfactant solutions, the use of salts to control the CMC, micelle size and shape, and the correlated viscosity makes them an important formulation adjunct for many industrial applications, such as home and personal care products. It is possible to rewrite eq 1 to describe the salt effect on the CMC of nonionic surfactants as^23^
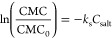
2where CMC and CMC_0_ are, respectively, the CMCs in aqueous salt solution and in pure water.

An attractive approach to surfactant formulation is to use computer simulations to supplement or even replace approximate theories and laborious experimental studies. Molecular dynamics (MD) has been extensively used to provide insights into the molecular details, for example, for protein–ion interactions of a single protein in a salt solution.^24−26^ Studies have been carried out on the effects of salt on the water structure,^27,28^ on the thermodynamics of hydration,^29^ and on molecular association.^21,30^ For example, Thomas and Elcock used MD to explore the correlation between the hydrophobic effect and water–ion hydrogen bonding using the Hofmeister series.^31^ However, statistically meaningful sample sizes are required, and the small size of the systems accessible using MD can be a problem for uncertainty quantification.^32^

Due to the high computational costs of MD studies of surfactants, coarse-grained (CG) approaches are more usually deployed to explore micelle formation in surfactant solutions.^33^ Salts have been incorporated in CGMD for both hard core and soft core repulsion methods.^34−41^ The MARTINI force field describes hard core repulsion using a shifted Lennard-Jones potential, where the parameters depend on the bead type and are optimized using experimental solubility data.^42,43^ Alternative methods for parameterization of soft core repulsion have recently been proposed.^37−39^ Here, we use dissipative particle dynamics (DPD), a soft core CGMD methodology, which has been developed quite extensively for surfactant simulations. DPD was first introduced by Hoogerbrugge and Koelman^44^ and later modified by Español and Warren^45^ to satisfy Gibbs–Boltzmann statistics in a canonical *NVT* ensemble.^46^ In DPD, a surfactant solution is modeled using soft particles called DPD beads. The solvent is represented by beads that represent two water molecules, salt ions are represented by adding charges to some of the water beads, and surfactant molecules are represented by a collection of connected DPD beads, which represent the various chemical subgroups. Dissipative and random forces between DPD beads provide a pairwise momentum-conserving thermostat,^45^ but the main pairwise interactions are soft, short-range repulsions derived from
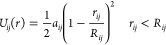
3where *a*_*ij*_ is the amplitude of the interaction between beads *i* and *j*, *r*_*ij*_ is the distance between the two beads, and *R*_*ij*_ represents the range of the interaction (cutoff distance). It is through the *a*_*ij*_ and *R*_*ij*_ parameters that chemical specificity is captured, and recent systematic approaches provide transferable DPD force fields for which the same set of parameters and fragmentation strategy can be used to generate a bead representation of different molecules in different environments. For this work, we use a recent DPD force field which has been extensively validated for surfactant simulations.^47,48^

In the case of added salt, as well as for ionic surfactants, electrostatic interactions must also be included. Methods to implement electrostatics in DPD have received much attention in recent years, with the most common approach being the Ewald method.^33,38,49−53^ However, in most studies, salt ions are represented simply as charged water beads,^37,54,55^ and very little work has been done to parameterize the (nonelectrostatic) short-range DPD repulsions with these charged beads despite this being an obvious target to capture ion-specific trends such as the Hofmeister series. Along these lines, Mayoral and Nahmad-Achar developed a parameterization for the repulsion amplitudes *a*_*ij*_ for the charged beads used in DPD based on the dependency of experimental Flory–Huggins χ parameters on salt concentration.^56^ More recently, Nieto-Draghi and Rousseau proposed a parameterization procedure for electrolytes in an aqueous solution based on osmotic pressure,^57^ but only ion–ion and ion–water bead interactions were investigated.

## Approach

A systematic strategy to parameterize the interactions between salt beads and the DPD beads which represent the surfactant chemistry has not yet been proposed. Here, we present such a method based on matching to experimental Setschenow coefficients. First, we identify the key interactions between salts and surfactants which affect the CMC. Second, we demonstrate that DPD simulations of surfactants show the same log-linear dependence of the CMC on salt concentration as that reported in eq 2, which allows us to define the DPD equivalents to the empirical Setschenow coefficients. In the third step, we correlate these Setschenow coefficients with the repulsion parameters. Then, in the final step, we utilize the DPD length-scale mapping to match with experimentally determined Setschenow coefficients, providing a systematic basis for fixing the DPD interaction parameters between ions and surfactant beads.

Speciation and micelle formation in aqueous solutions is one of the most common targets of DPD surfactant simulations.^33,58^ As already described, treating ions as charged water beads is a simple and convenient approach that has been used to obtain qualitatively good results for several systems.^38,39^ However, this approach assumes that all ions are the same, which is in contrast to the experimental evidence for ion-specific trends. Table 1 shows that the experimental Setschenow coefficients that describe the effects of different salts on four nonionic surfactants, *n*-octanoyl-*N*-methylglucamine (MEGA8), *n*-nonanoyl-*N*-methylglucamine (MEGA9), *n*-octyl glucopyranoside (GLUCO8), and hexaethylene glycol monododecyl ether (C12E6), cover a wide range of values.

**Table 1:** Experimental Setschenow Coefficients (*k*_s_) for Nonionic Surfactants in Units of per Mole

	surfactants			
salts	MEGA8^57^	MEGA9^57^	GLUCO8^58^	C12E6^59^
LiCl	0.33	0.36	0.43	0.53
NaF	0.87	1.01		
NaCl	0.53	0.56	0.57	0.81
NaBr	0.37	0.45		
NaNO_3_	0.30	0.37		
NaI	0.35	0.35		
NaSCN	0.29			
KCl	0.47	0.56		0.69
KBr	0.34	0.38		0.48
KI	0.26	0.30		0.30
CsCl	0.39			
CaCl_2_	0.67			
Na_2_HPO_4_	1.20			
Na_2_SO_3_	1.38			
Na_2_CO_3_	1.53	1.79		
Na_2_SO_4_	1.59	1.85		

**Figure 1:**
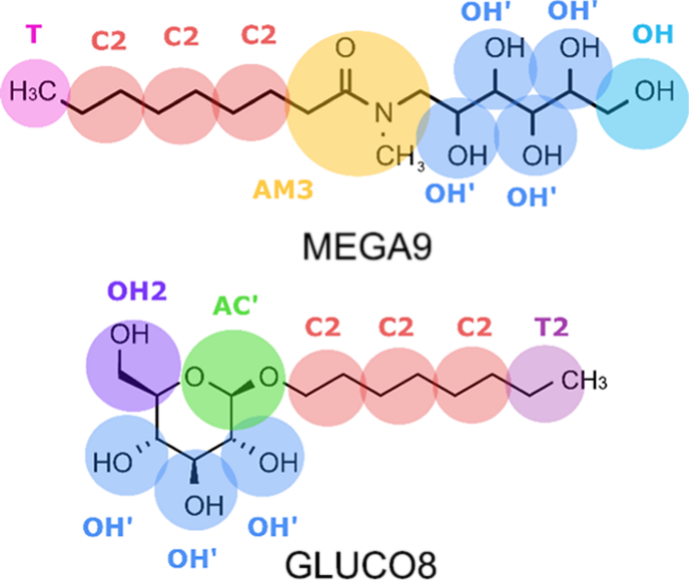
CG representations of MEGA9 and GLUCO8.

We selected two nonionic surfactants to use for initial parameterization.^60,61^ DPD simulations perform most reliably for CMC values in the range 1–100 mM: if the CMC is too high, there is no distinction between submicellar aggregates and true micelles because of the overlap between the submicellar and micellar populations, and conversely, if the CMC is too low, the small number of free surfactants in solution leads to uncertainty.^38,47,62^ Accordingly, we selected GLUCO8 and MEGA9, and the CG representations of these surfactants are shown in Figure 1. Table 1 includes Setschenow coefficients for some divalent ions (calcium, monohydrogen phosphate, sulfate, and carbonate). We include these for completeness since they may be useful in future work. To avoid complications arising from the nonideality of salt solutions containing multivalent ions, which may be significant, the present study focuses on the monovalent ions in Table 1.

## Simulation Details

DPD simulations were run for all the surfactant systems at different salt concentrations. The CG representation of the surfactants and the repulsion parameters for the beads were obtained from previous studies (Figure 1 and Table 2).^47,48^ The cutoff distances in Table 2 were assigned as given in Anderson *et al.*(63) The repulsion amplitude between water beads is chosen conventionally as 25 *k*_B_*T* so that the pressure of pure water in DPD units is 23.7.^63^ In the model, the cutoff distance *R*_*ij*_ between water beads is defined as the DPD unit of length *r*_c_, pure water is represented by water beads at a reduced density ρ*r*_c_^3^ = 3, and we suppose that each water bead represents *N*_m_ water molecules, where *N*_m_ is the so-called (water bead) mapping number.^64^ To fit with the chosen DPD force field, we use *N*_m_ = 2.^45,62^ If *V*_m_ ≈ 18 × 10^–6^ m^3^ is the molar volume of water, one can deduce that the volume of 1 mol of DPD volume elements *N*_A_*r*_c_^3^ = ρ*r*_c_^3^ × *N*_m_ × *V*_m_ ≈ 0.108 × 10^–3^ m^3^ ≈ 0.108 litres, and hence, *r*_c_ = 5.64 Å. The DPD volume can be used to convert the number of salt beads (*N*_salt_) to molar concentration units; namely, if the simulation box side is *L*, then
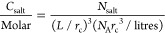
4

**Table 2:** DPD Parameters for All Pairwise Bead Interactions (Repulsion Amplitudes in Units of *k*_B_*T* and Cutoff Distances in Units of *r*_c_)

bead1	bead2	*a*_*ii*_	*a*_*ij*_	Δ*a*_*ij*_	*R*_*ij*_
C2	C2	22.0			1.074
EO	EO	25.5			1.116
OH	OH	14.0			0.980
OH′	OH′	14.0			0.949
W	W	25.0			1.000
T	T	24.0			0.955
T2	T2	24.0			1.098
AC′	AC′	22.5			0.952
AM3	AM3	22.0			1.296
OH2	OH2	18.0			1.012
C2	EO		23.78	0.03	1.095
C2	OH		27.13	9.13	1.027
C2	W		45.54	21.95	1.037
C2	OH′		28.77	10.77	1.012
C2	AM3		21.83	–0.17	1.185
C2	AC′		18.17	–4.08	1.013
C2	T2		21.97	–1.03	1.086
OH′	AM3		11.00	–7.00	1.123
OH′	OH		13.86	–0.14	0.965
OH′	OH2		15.95	–0.05	0.981
OH′	AC′		19.42	1.17	0.951
OH′	T		28.85	9.85	0.952
OH′	T2		28.28	9.28	1.024
OH′	W		15.09	–4.41	0.975
EO	OH		18.17	1.33	1.048
EO	W		21.81	3.44	1.058
OH	W		18.17	1.33	0.990
OH	AM3		11.52	–6.48	1.138
OH2	AC′		17.42	–2.83	0.982
T	W		46.35	21.85	0.978
T	OH		27.49	8.49	0.968
T	C2		22.92	0.08	1.015
T	EO		24.18	1.05	1.036
T	AM3		22.32	–0.68	1.126
W	OH2		22.20	0.70	1.006
W	AC'		7.74	–16.01	0.976
W	AM3		13.20	–10.30	1.148
W	T2		45.44	20.94	1.049
T2	AC′		17.32	–5.93	1.025
T2	OH2		27.59	6.59	1.024

Molecularly bonded beads are held together with a harmonic spring potential
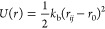
where *k*_b_ = 150 *k*_B_*T*, and a three-body angular potential
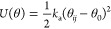
where *k*_a_ = 5 *k*_B_*T*. The equilibrium distance *r*_0_ and the equilibrium angle θ_0_ were assigned using the method previously reported.^48^

Simulations were performed in a cubic box of side *L* = 30 *r*_c_ with the total number of beads equal to 81,000. They were run for 4 × 10^6^ steps with a time step of 0.01 in DPD time units, starting from a random initial configuration. By measuring the diffusion of small molecules in a related DPD model with a comparable level of coarse graining, Sevink and Fraaije determined the underlying DPD time unit ≈ 50 ps, so the time step in our simulations should correspond to about 0.5 ps,^65^ making the total simulation run time 1–2 μs. This timescale is long enough for micelles to form and to equilibrate. Simulations were run in the isothermal–isobaric ensemble (*NPT*) using the standard velocity Verlet integration algorithm.^66^ Trajectory files were collected every 10^3^ time steps. Simulations were performed using DL_MESO (version 2.7).^67^ Post-simulation trajectory analysis was performed using a combination of the UMMAP analysis tool^68^ and bespoke analysis scripts.

DPD simulations were run at 4, 5, and 6 wt % for all surfactants. The aggregation number distribution is a plot of population *P*(*N*) *versus* aggregation number (*N*), and this distribution can be used to discriminate between monomers and submicellar aggregates (designated free surfactant) and stable micelles as described in previous studies.^47,48^ By plotting this distribution, one discerns a region depleted in stable micelles, which allows the definition of a value of *N*_cut_ to separate the free surfactants (*N* < *N*_cut_) from micelles (*N* > *N*_cut_). For each simulation, the minimum in *P*(*N*) from the aggregation number distribution was used as *N*_cut_. The CMC was calculated as the total concentration of free surfactants after reaching the equilibrium (typically after 5 × 10^5^ steps). The values of CMC did not vary significantly with surfactant concentration, and the average values are quoted.

As a starting point, each salt ion was represented by a positive bead or a negative bead, with the same *R*_*ij*_ and *a*_*ij*_ as that of water (*i.e.*, as charged water beads), and then, the *a*_*ij*_ values were varied to study the effect on the calculated CMC of the surfactant. The Slater-type charge smearing for the electrostatic interactions proposed by González-Melchor *et al.* was adopted.^69^ For a pair of particles, the electrostatic interaction potential can be written as
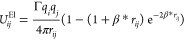
5where *r*_*ij*_ is the distance between particles *i* and *j*, *q*_*i*_ and *q*_*j*_ are the ion valences, and β* is the Slater smearing parameter (set to be equal to 0.929 *r*_c_^–1^). The strength of the interaction is governed by Γ = e^2^/(*k*_B_*T*ε_0_ε_r_*r*_c_), which is a dimensionless electrostatic coupling parameter. Following Vaiwala *et al.*(70) and Anderson *et al.*,^38^ we assume a uniform relative dielectric permittivity of ε_r_ = 78.3 and *T* = 298 K, resulting in Γ = 15.94 for *r*_c_ = 5.64 Å. The *k*-vector cutoff in the *k*-space was set as 5 *r*_c_^–1^. Changing the cutoff from 1 *r*_c_^–1^ to 10 *r*_c_^–1^ did not show any effect on the CMC value of neutral surfactants. The real-space Ewald cutoff was set as 3.0 *r*_c_.

## Results and Discussion

**Figure 2:**
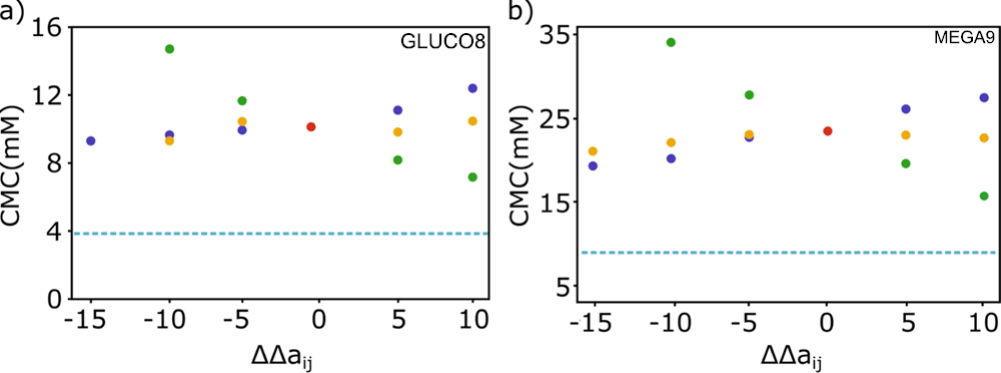
Calculated CMC value plotted as a function of the change in the repulsion parameter Δ*a*_*ij*_ relative to the water–water value for interactions of the ion beads with water (blue), hydrophilic head groups (yellow), and the hydrophobic tail (green) for (a) GLUCO8 and (b) MEGA9. The red point represents the CMC values obtained using the standard water *a*_*ij*_ values (*i.e.*, Δ*a*_*ij*_ = 0). The salt concentration was 1 M, and the dotted line shows the experimental CMC value obtained for the surfactant in a 1 M solution of NaCl.

First, the effect of changing the values of *a*_*ij*_ for interactions between salt ions and other beads was investigated though the effect on the CMC. Figure 2 shows the relationship between the CMC and the *a*_*ij*_ parameters for ion–water (blue), ion–tail (green), and ion–head group (yellow) interactions. Beads T, T2, and C2 were considered part of the hydrophobic tail, and beads AM3, OH, OH′, OH2, and AC′ were considered part of the hydrophilic head group. Figure 2 compares the results for MEGA9 and GLUCO8 obtained using a salt concentration of 1 M with the experimental values (dotted line). The plots show that the ion–tail interactions have a much bigger effect in decreasing the CMC values than the ion–head and ion–water interactions and that the best way to approach values closer to the experimental values is by further increasing the repulsion parameter for the ion–tail interactions. Therefore, we focused our attention on these interaction parameters, which is in line with the conclusions obtained by Mukerjee in his studies on C_*x*_E_*y*_ surfactants, where the contribution of the hydrophilic head to *k*_s_ for neutral surfactants was reported to be negligible.^23^

**Figure 3:**
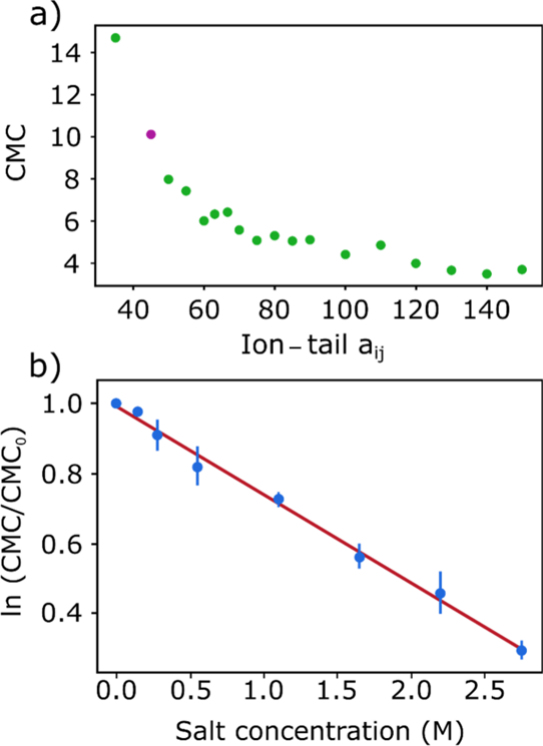
(a) Relationship between the CMC value and the ion–tail *a*_*ij*_ parameter for GLUCO8. (b) Linear correlation between ln(CMC/CMC_0_) and salt concentration for *a*_*ij*_ = 100 *k*_B_*T*.

Ion–tail *a*_*ij*_ values were screened from 35 *k*_B_*T* to 150 *k*_B_*T* for both the anion (*a*_*ij*^–^_) and cation (*a*_*ij*^+^_), and the CMC values were calculated for both surfactants. The result for GLUCO8 with *a*_*ij*^+^_ = *a*_*ij*^–^_ reported in Figure 3a shows a nonlinear relationship, which reaches a plateau around *a*_*ij*_ = 140 *k*_B_*T*. The CMC value increases sharply for small *a*_*ij*_ values, which is consistent with the negative *k*_s_ values observed for polyoxyethylenes.^71^ The effect of salt concentration on the CMC value was then studied. The CMC values were averaged from three independent simulation runs with surfactant concentrations of 4, 5, and 6 wt %. These simulations were repeated using different ion–tail *a*_*ij*_ values and for a range of salt concentrations between 0 and 2.75 M. Figure 3b shows an example of the results. In all cases, a clear log-linear relationship was found between CMC and salt concentration.

**Figure 4:**
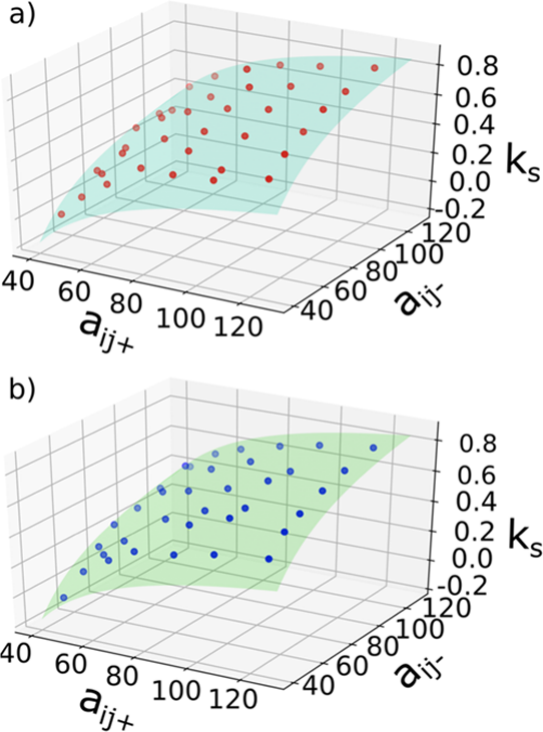
Relationship between the values of *k*_s_ obtained from DPD simulations and the repulsion parameters for the cation–tail (*a*_*ij*^+^_) and anion–tail (*a*_*ij*^–^_) interactions for (a) MEGA9 and (b) GLUCO8. The shaded surfaces were obtained by fitting to eq 6.

Slopes of plots of the CMC values obtained in DPD simulations *versus* salt concentration were used to calculate values of *k*_s_ as a function of the ion–tail repulsion parameters used in the simulations. The relationship between the calculated values of *k*_s_ and the values of *a*_*ij*^–^_ and *a*_*ij*^+^_ for the anion–tail and cation–tail interactions is shown in Figure 4 for GLUCO8 and MEGA9. The surfaces colored in green in Figure 4 are the best fit for the following relationship
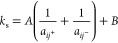
6where *A* and *B* are constants that depend on the surfactant (Table 3).

**Table 3:** *A* and *B* Parameters Used to Describe Different Surfactants in Eq 6^a^

	surfactants			
parameter	MEGA8	MEGA9	GLUCO8	C12E6
*A*	–29.42	–30.03	–29.04	–52.32
*B*	1.24	1.32	1.29	2.09

a Sum of the square of residuals from fitting: MEGA8 0.00053, MEGA9 0.00037, C12E6 0.00123, and GLUCO8 = 0.00067 M^–1^.

**Figure 5:**
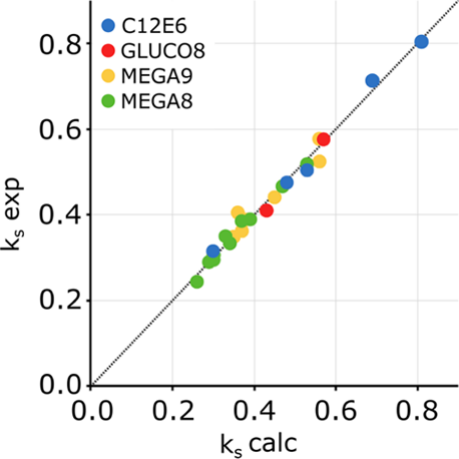
Comparison of the calculated values of *k*_s_ with the experimental values for four different surfactants in 10 different salt solutions (in blue, C12E6; in red, GLUCO8; in yellow, MEGA9; and in green, MEGA8). *R*^2^ = 0.98.

The interesting property of eq 6 is that the terms that describe interactions with the anion and the cation appear separately so that a salt can be described simply as the sum of the individual effects of the anion and the cation on the CMC. Having established values of *A* and *B* for GLUCO8 and MEGA9, the experimentally determined values of *k*_s_ measured for these surfactants in different salt solutions can be used in conjunction with eq 6 to derive the repulsion parameters required to describe the individual ions. The ion–tail repulsion parameters for lithium, sodium, and potassium cations and for chloride, bromide, and iodide anions were obtained by fitting the experimental values of *k*_s_ for GLUCO8 and MEGA9 in Table 1 to eq 6 using a generalized reduced gradient nonlinear method. Having established repulsion parameters for a range of different anions and cations, the experimentally determined values of *k*_s_ measured for different surfactants in the corresponding salt solutions can be used in conjunction with eq 6 to derive the constants *A* and *B* required to describe the surfactants. The parameters for MEGA8 and C12E6 were obtained by fitting the experimental values of *k*_s_ in Table 1 to eq 6, and the results are shown in Table 3. The repulsion parameters for the ions listed in Table 4 were then optimized by using the experimental values of *k*_s_ for all four surfactants in eq 6 and the values of *A* and *B* in Table 3. The parameters in Tables 3 and 4 provide an excellent description of the experimental *k*_s_ values for all four surfactants in solutions of 10 different salts (Figure 5). The results suggest that eq 6 can be used to predict the effects of salts on other surfactants, provided sufficient experimental data are available to estimate the *A* and *B* parameters for the surfactant (Table 5).

**Table 4:** Ion–Tail Repulsion Parameters for Cations and Anions

cation	anion	*a*_*ij*^+^_	*a*_*ij*^–^_
Li^+^		51	
Na^+^		72	
K^+^		64	
Cs^+^		55	
	Cl^–^		94
	Br^–^		66
	I^–^		55
	NO_3_^–^		56
	SCN^–^		54

**Table 5:** Comparison of Calculated and Experimental Setschenow Coefficients (*k*_s_) in Units of per Mole

surfactant	cation	anion	*k*_s_ exp	*k*_s_ calc	error
C12E6	Li^+^	Cl^–^	0.53	0.50	0.03
	Na^+^	Cl^–^	0.81	0.80	0.01
	K^+^	Cl^–^	0.69	0.71	–0.02
	K^+^	Br^–^	0.48	0.48	0.00
	K^+^	I^–^	0.30	0.32	–0.02
GLUCO8	Na^+^	Cl^–^	0.57	0.58	–0.01
	Li^+^	Cl^–^	0.43	0.41	0.02
MEGA8	Li^+^	Cl^–^	0.33	0.35	–0.02
	Na^+^	Cl^–^	0.53	0.52	0.01
	Na^+^	Br^–^	0.37	0.39	–0.02
	Na^+^	NO_3_^–^	0.30	0.31	–0.01
	Na^+^	I^–^	0.30	0.30	0.00
	Na^+^	SCN^–^	0.29	0.29	0.00
	K^+^	Cl^–^	0.47	0.47	0.00
	K^+^	Br^–^	0.34	0.33	0.01
	K^+^	I^–^	0.26	0.24	0.02
	Cs^+^	Cl^–^	0.39	0.39	0.00
MEGA9	Li^+^	Cl^–^	0.36	0.41	–0.05
	Na^+^	Cl^–^	0.56	0.58	–0.02
	Na^+^	Br^–^	0.45	0.44	0.01
	Na^+^	NO_3_^–^	0.37	0.36	0.01
	Na^+^	I^–^	0.35	0.35	0.00
	K^+^	Cl^–^	0.56	0.52	0.04
	K^+^	Br^–^	0.38	0.39	–0.01
	K^+^	I^–^	0.30	0.30	0.00

The parameters for thiocyanate and caesium ions in Table 4 were obtained from single measurements and should be considered less reliable than the other values. The parameter for the fluoride ion could not be determined by changing the ion–tail repulsion parameter because the *a*_*ij*_ value required was too large. It is clear that fluoride has the highest repulsion parameter of all of the anions, but some other interactions must be involved to account for the behavior of these systems, such as a specific interaction with the surfactant or a direct effect on the counterion. The trends in *a*_*ij*_ values are F^–^ > Cl^–^ > Br^–^ > NO_3_^–^ > I^–^ > SCN^–^ for anions and Na^+^ > K^+^ > Cs^+^ > Li^+^ for cations. With the exception of K^+^, which precedes Na^+^ in the cation series, both sequences match the Hofmeister series.^1,72^

## Conclusions

A method for calculating the interaction parameters between salt ion beads and surfactant beads for DPD simulations has been developed. DPD simulations show that the calculated CMC values depend largely on the ion–tail repulsion parameter, and to a first approximation, the other interactions in the system can be ignored. When CMC values were calculated as a function of the concentration of salt ion beads, the results were found to reproduce the empirical Setschenow relationship, and the calculated values of the Setschenow constant *k*_s_ therefore provide a direct connection with an experimentally determined parameter that describes the interaction of ions with surfactants in aqueous salt solutions. A general equation has been derived that describes the Setschenow constant *k*_s_ in terms of the repulsion parameter for the cation–tail interaction, the repulsion parameter for the anion–tail interaction, and two surfactant parameters *A* and *B*. By fitting the calculated values of *k*_s_ to the experimental values, it was possible to derive *A* and *B* parameters for four different surfactants and repulsion parameters for a range of different ions. The resulting parameters provide accurate descriptions the experimental behavior of these surfactant systems in 10 different salt solutions. This result provides a general approach for the parameterization of repulsion parameters for charged species in DPD simulations.

Our observation that the Setschenow trends can be captured in the present model by tuning the ion–tail interactions supports the notion that the Setschenow coefficients are a measure of the solvent “quality” in these systems. Thus, our results underscore the idea that, in this context, the Hofmeister series reflects changes in the hydrophobic effect acting on the surfactant tails.^73^ We emphasize in this respect that apart from the ion–tail interactions in Table 4, the ions are otherwise treated as charged water beads (*cf*., the “W” beads given in Table 2). This means for instance that trends in ion activities are *not* reproduced in the present model. This could be solved by combining the approach with, for example, the parameterization strategy for ion–ion and ion–water interactions proposed by Nieto-Draghi and Rousseau,^57^ which could then be extended to include multivalent ions. Further work will also be required to extend the approach to ionic surfactants and to test the effect of changes in the nature of the hydrocarbon tail. The case of polyions (polyelectrolytes) is also of considerable interest; however, these are often involved with very specific effects such as adsorption onto surfactant micelles^74^ and would require separate treatment.
